# Associação da Osmolalidade Plasmática com o Risco de Doença Cardiovascular: Evidências do Banco de Dados do National Health and Nutrition Examination Survey

**DOI:** 10.36660/abc.20250176

**Published:** 2025-12-08

**Authors:** Feng Gao, Yicheng Shi, Yi Chen, Shu Lu

**Affiliations:** 1 Nanjing University of Traditional Chinese Medicine Wuxi Hospital Cardiovascular Wuxi China Nanjing University of Traditional Chinese Medicine Affiliated Wuxi Hospital – Cardiovascular, Wuxi – China

**Keywords:** Doenças Cardiovasculares, Insuficiência Cardíaca, Doença das Coronárias, Infarto do Miocárdio

## Abstract

**Fundamento:**

A doença cardiovascular (DCV) continua sendo a principal causa de morte no mundo. Diante de seu elevado impacto em saúde pública, ainda são necessários estudos que definam fatores de risco ao mesmo tempo modificáveis e mensuráveis.

**Objetivo:**

Investigar a associação entre a osmolalidade plasmática (Osm) e DCV — especificamente insuficiência cardíaca, doença coronariana, angina e infarto do miocárdio — utilizando dados do National Health and Nutrition Examination Survey (NHANES).

**Métodos:**

Foram analisados nove ciclos do NHANES (1999-2018). As associações entre Osm e DCV foram avaliadas por regressão logística ponderada. Estimaram-se razões de chances (OR) e IC 95%, além de análises por subgrupos. Empregaram-se modelos *restricted cubic spline* (RCS) para investigar possíveis relações não lineares. Adotou-se significância estatística de p < 0,05.

**Resultados:**

O aumento de Osm associou-se a maiores chances de insuficiência cardíaca, doença coronariana, angina e infarto do miocárdio nos modelos não ajustados e parcialmente ajustados. Após o ajuste completo, as associações permaneceram significativas para todos os desfechos, exceto angina. As análises com RCS indicaram relação em U significativa para insuficiência cardíaca e angina em todos os modelos, com menor risco em torno de 278 mmol/kg. As análises de subgrupos foram consistentes com os resultados principais.

**Conclusão:**

O aumento de Osm associa-se de forma significativa ao risco de DCV. A Osm pode ser um biomarcador útil para avaliação e manejo do risco cardiovascular na prática clínica, sujeito à confirmação em estudos de coorte prospectivos.

## Graphical abstract

**Figura Central:**
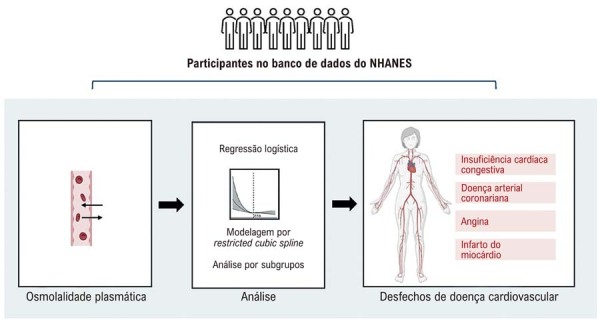
Associação da Osmolalidade Plasmática com o Risco de Doença Cardiovascular: Evidências do Banco de Dados do National Health and Nutrition Examination Survey

**Figura Central:** Associação da Osmolalidade Plasmática com o Risco de Doença Cardiovascular: Evidências do Banco de Dados do National Health and Nutrition Examination Survey

NHANES: National Health and Nutrition Examination Survey.

## Introdução

A doença cardiovascular (DCV) abrange condições que afetam o coração e os vasos sanguíneos, incluindo doença arterial coronariana (DAC), hipertensão e insuficiência cardíaca (IC).^1 - 5^ A Organização Mundial da Saúde reconhece a DCV como a principal causa de morte no mundo, responsável por cerca de 17,9 milhões de óbitos por ano,^6^ com impactos profundos sobre pacientes e famílias e um substancial ônus econômico para a sociedade. Apesar dos avanços nas terapias farmacológicas, intervencionistas e cirúrgicas, persistem limitações importantes — especialmente na estratificação de risco e no tratamento personalizado.^5 , 7 - 9^ Há, portanto, uma necessidade urgente de pesquisas que aprofundem a compreensão dos fatores de risco para DCV.

A osmolalidade plasmática (Osm) regula o equilíbrio hídrico entre os compartimentos intra- e extracelular e entre os espaços intra- e extravascular, sendo amplamente determinada por Na^+^, K^+^ e Cl^–^. A Osm é calculada como: $\text{ Osm }=2\times {\mathrm{Na}}^{+}+\text{qlicose/18 + nitrogênio ureico no sangue (BUN)2,8}$ , com faixa normal de 275-295 mOsm/kg.^10^ Medir e monitorar a Osm é essencial na avaliação e no manejo de diversas condições. Alterações de Osm relacionam-se estreitamente à evolução de distúrbios como hiponatremia, hipernatremia, hiperglicemia, osteoartrite, nefropatia e doença pulmonar obstrutiva crônica.^11^ Evidências emergentes também sugerem relação entre Osm e desfechos cardiovasculares. Em pessoas com diabetes, tanto valores baixos quanto elevados de Osm associaram-se ao aumento da mortalidade por todas as causas, indicando uma associação em U.^12^ A razão BUN/albumina (BAR) — sendo o BUN um dos principais componentes da Osm — prediz mortalidade em DAC e insuficiência cardíaca congestiva (ICC) graves.^13 , 14^ Além disso, a Osm é um importante indicador prognóstico em ICC, correlacionando-se com a mortalidade por todas as causas em 28 dias.^15^ No entanto, a maior parte dos estudos prévios concentrou-se no prognóstico de pacientes com DCV estabelecida, e ainda são limitadas as avaliações abrangentes da relação entre Osm e risco de DCV na população geral.

Utilizando dados do National Health and Nutrition Examination Survey (NHANES), examinamos a associação entre Osm e o risco de ICC, DAC, angina e infarto do miocárdio, com o objetivo de informar a avaliação clínica de risco.

## Métodos

### Participantes do estudo

Utilizamos dados do NHANES (https://www.cdc.gov/nchs/nhanes/index.html), um programa nacionalmente representativo que avalia o estado de saúde e nutricional da população dos EUA por meio de amostragem probabilística contínua em múltiplos estágios.^16^ As entrevistas domiciliares foram seguidas de exames físicos e laboratoriais realizados em Centros de Exames Móveis (CEMs). Todos os protocolos do NHANES foram aprovados pelo National Center for Health Statistics Ethics Review Board, e todos os participantes assinaram termo de consentimento livre e esclarecido. Todas as análises seguiram as diretrizes e regulamentações do NHANES.

Foram analisados os nove ciclos mais recentes (1999-2018), totalizando 91.351 participantes. Indivíduos sem mensuração de Osm ou sem dados do questionário de DCV foram excluídos.

### Exposição e desfechos

A Osm foi medida nos CEMs usando a variável do NHANES LBXSOSSI, com variação de 201 a 323 mmol/kg. Os participantes foram categorizados em quartis (Q1-Q4), sendo Q1 o menor e Q4 o maior valor de Osm.

Os desfechos primários foram as prevalências de ICC, doença arterial coronariana (DAC), angina e infarto do miocárdio, definidos por autorrelato no questionário: MCQ160b (“Alguma vez lhe disseram que teve ICC?”), MCQ160c (“Alguma vez lhe disseram que teve DAC?”), MCQ160d (“Alguma vez lhe disseram que teve angina, angina pectoris?”) e MCQ160e (“Alguma vez lhe disseram que teve ataque cardíaco?”). As respostas “Sim” classificaram o participante como portador da condição; respostas “Não” indicaram ausência da condição.

### Covariáveis

Seguindo Bays et al.,^17^ ajustamos por: idade; sexo; raça/etnia (mexicano-americano, outro hispânico, branco não hispânico, negro não hispânico, outros); histórico de hipertensão e diabetes; pressão arterial sistólica (PAS) e diastólica (PAD); tabagismo; índice de massa corporal (IMC); triglicerídeos (TG); colesterol de lipoproteína de baixa densidade (LDL-C); BUN; e glicose sanguínea. Características demográficas e históricos de hipertensão, diabetes e tabagismo foram obtidos por questionário. SBP, DBP e BMI derivaram dos exames realizados nos CEMs; TG, LDL, BUN e glicose vieram dos dados laboratoriais dos CEMs. O status tabágico foi categorizado como “Todos os dias”, “Alguns dias” ou “Não fuma”. TG foi dicotomizado em ≤ 1,70 vs. > 1,70 mmol/L, e LDL em ≤ 3,37 vs. > 3,37 mmol/L. Para reduzir viés por dados ausentes, variáveis com ≤ 20% de *missing* foram imputadas por múltipla imputação; para variáveis com > 20% de *missing* , criaram-se categorias incluídas como variáveis *dummy* nos modelos.

### Análise estatística

A normalidade foi avaliada pelo teste de Shapiro-Wilk. Como todas as variáveis contínuas apresentaram distribuição não normal, foram reportadas como medianas e intervalos interquartis e comparadas pelo teste de Mann-Whitney *U* . Variáveis categóricas são apresentadas como contagens (n) e porcentagens (%) e comparadas pelo teste do qui-quadrado.

A associação entre exposição e desfechos foi estimada por regressão logística ponderada (RLP), com inclusão sequencial de covariáveis. Modelo 1: sem ajustes; Modelo 2: ajustado por idade, sexo e raça; Modelo 3: adicionalmente ajustado por hipertensão, diabetes, tabagismo, PAS, PAD, IMC, TG, LDL, BUN e glicose sanguínea. Para avaliar o desempenho discriminativo do Modelo 3, geramos curvas *receiver operating characteristic* (ROC) e calculamos a *area under the curve* (AUC) e o pseudo-R^2^ de McFadden. Para mitigar multicolinearidade, computamos os fatores de inflação da variância (FIV) e excluímos variáveis com FIV > 5. Modelos RCS avaliaram possíveis relações não lineares. Em seguida, realizaram-se análises de subgrupos, apresentadas como *forest plots* . As análises foram conduzidas no R versão 4.4.2, com p bicaudal < 0,05 considerado estatisticamente significativo.

## Resultados

**Figura 1:**
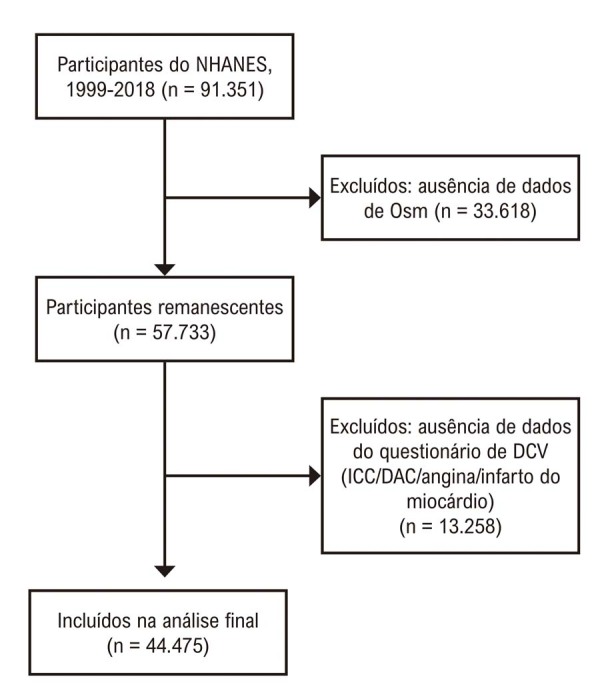
– Fluxograma de inclusão do estudo. DCV: doença cardiovascular; DAC: doença arterial coronariana; ICC: insuficiência cardíaca congestiva; NHANES: National Health and Nutrition Examination Survey; Osm: osmolalidade plasmática.

No total, 44.475 participantes atenderam aos critérios de elegibilidade ( Figura 1 ). Como todas as variáveis contínuas apresentaram distribuição não normal, utilizou-se o teste de Mann–Whitney U. As características basais ( Tabela 1 ) indicam predomínio de mulheres, participantes brancos não hispânicos e indivíduos ≤ 60 anos. Em comparação com Q1 (menor Osm), Q4 (maior Osm) incluiu mais homens e adultos mais velhos (> 60 anos) e apresentou maiores prevalências de hipertensão, diabetes, ICC, DAC, angina e infarto do miocárdio, além de valores mais altos de IMC, PAS, BUN e glicose.

**Tabela 1:** – Características basais dos participantes por quartil de Osm

Característica	Total	Q1	Q2	Q3	Q4	Valor p
**N (ponderado)**	206.406.933	58.579.788	53.539.459	58.183.076	36.104.609	
**IMC**	28 [24-32]	27 [24-32]	27 [24-32]	28 [24-32]	29 [25-33]	<0,001***
**Hipertensão, n (%)**	15.455 (31)	3.205 (25)	3.112 (26)	4.552 (32)	4.586 (46)	<0,001***
**PAS, mmHg**	120 [112-132]	118 [110-128]	118 [110-130]	122 [112-132]	126 [116-136]	<0,001***
**PAD, mmHg**	71 [64-78]	70 [64-78]	71 [64-78]	72 [64-78]	71 [64-78]	0,028*
**Sexo (%)**						<0,001***
Homens	21.482 (48)	4.562 (37)	5.391 (49)	6.753 (55)	4.776 (54)	
Mulheres	22.993 (52)	7.895 (63)	5.440 (51)	5.595 (45)	4.063 (46)	
**Faixa etária (%)**						<0,001***
< 40 anos	15.265 (37)	6.213 (49)	4.379 (42)	3.460 (32)	1.213 (19)	
40-60 anos	15.300 (40)	4.067 (38)	4.020 (42)	4.502 (41)	2.711 (37)	
> 60 anos	13.910 (23)	2.177 (13)	2.432 (16)	4.386 (27)	4.915 (45)	
**Raça/Etnia (%)**						<0,001***
Mexicano-americano	7.499 (8,3)	2.238 (8,9)	1.880 (8,7)	2.030 (7,8)	1.351 (7,7)	
Outro hispânico	3.753 (5,4)	981 (5,5)	987 (5,8)	1.045 (5,1)	740 (5,2)	
Negro não hispânico	9.069 (11)	2.639 (12)	2.195 (10)	2.402 (9.7)	1.833 (10)	
Branco não hispânico	19.835 (68)	5.397 (66)	4.711 (68)	5.678 (70)	4.049 (69)	
Outros	4.319 (7,1)	1.202 (7,2)	1.058 (7,1)	1.193 (6,9)	866 (7,2)	
**Status de diabetes (%)**						<0,001***
Sim	5.403 (9,0)	697 (4,3)	816 (5,8)	1.532 (9,3)	2.358 (21)	
Não	38.140 (89)	11.581 (94)	9.827 (93)	10.532 (89)	6.200 (76)	
Limítrofe	932 (1,9)	179 (1,3)	188 (1,5)	284 (2,0)	281 (3.2)	
**Tabagismo (%)**						<0,001***
Todos os dias	7.531 (17)	2.505 (22)	1.985 (18)	1.968 (15)	1.073 (12)	
Ausente	24.275 (54)	6.917 (53)	6.033 (56)	6.648 (54)	4.677 (54)	
Não fuma	10.939 (25)	2.491 (21)	2.321 (22)	3.293 (27)	2.834 (31)	
Alguns dias	1.730 (3,7)	544 (4,2)	492 (4,2)	439 (3,4)	255 (2,8)	
**Triglicerídeos, n (%)**						0,017*
≤ 1.70 mmol/l	15.482 (35)	4.199 (34)	3.880 (35)	4.308 (35)	3.095 (36)	
> 1.70 mmol/l	5.877 (13)	1.618 (13)	1.366 (12)	1.636 (13)	1.257 (14)	
Ausente	23.116 (52)	6.640 (54)	5.585 (52)	6.404 (52)	4.487 (50)	
**LDL-C (%)**						<0,001***
≤ 3.37 mmol/l	14.486 (33)	3.990 (32)	3.526 (32)	3.913 (32)	3.057 (35)	
> 3.37 mmol/l	6.271 (14)	1.643 (13)	1.598 (14)	1.876 (15)	1.154 (14)	
Ausente	23.718 (53)	6.824 (55)	5.707 (54)	6.559 (53)	4.628 (51)	
**BUN, mg/dl**	13,0 [10,0-16,0]	11,0 [8,0-13,0]	12,0 [10,0-15,0]	14,0 [11,0-17,0]	16,0 [13,0-21,0]	<0,001***
**Glicose plasmática, mmol/l**	5,05 [4,72, 5,61]	4,88 [4,55, 5,27]	5,00 [4,66, 5,44]	5,16 [4,77, 5,72]	5,50 [5,00, 6,61]	<0,001***
**ICC, n (%)**	1.397 (2,3)	216 (1,4)	200 (1,3)	363 (2,2)	618 (5,3)	<0,001***
**DAC, n (%)**	1.815 (3,4)	275 (1.8)	294 (2,3)	511 (3,6)	735 (7,5)	<0,001***
**Angina, n (%)**	1.218 (2,3)	215 (1.6)	198 (1,5)	355 (2,3)	450 (4,7)	<0,001***
**Infarto do miocárdio, n (%)**	1.856 (3,2)	294 (2.0)	292 (2,1)	555 (3,5)	715 (6,5)	<0,001***

Dados apresentados como mediana [Q1, Q3] ou n (%). Quartis de osmolalidade plasmática (mOsm/kg): Q1 201-275; Q2 275-278; Q3 278-282; Q4 282-323. N (ponderado) indica o tamanho amostral ponderado. Limiar de valores de p: *p < 0,05; **p < 0,01; ***p < 0,001. BUN: nitrogênio ureico; DAC: doença arterial coronariana; ICC: insuficiência cardíaca congestiva; IMC: índice de massa corporal; LDL-C: colesterol de lipoproteína de baixa densidade; PAD: pressão arterial diastólica; PAS: pressão arterial sistólica; Osm: osmolalidade plasmática.

As análises por RLP ( Tabela 2 ) mostraram que Osm mais elevada associou-se ao aumento substancial do risco de DCV nos modelos não ajustado, parcialmente ajustado e totalmente ajustado. Quando modelada como variável contínua, Osm mais alta associou-se significativamente a maiores riscos de ICC, DAC e infarto do miocárdio nos três modelos. Para angina, observou-se o mesmo padrão nos modelos não ajustado e parcialmente ajustado, mas a associação deixou de ser significativa após o ajuste completo ( Tabela 2 ).

**Tabela 2:** – Associação entre Osm e desfechos cardiovasculares

Característica	Modelo 1			Modelo 2			Modelo 3		
	OR	IC 95%	Valor p	OR	IC 95%	Valor p	OR	IC 95%	Valor p
**DAC**									
Osm (contínua)	1,116	1,097-1,135	<0,001	1,059	1,042-1,076	<0,001	1,033	1,017-1,049	<0,001
Osm (categórica)	Valor de p para tendência < 0,001			Valor de p para tendência < 0,001			Valor de p para tendência = 0,004		
Q1	—	—		—	—		—	—	
Q2	0,946	0,745-1,201	0,645	0,816	0,642-1,037	0,096	0,825	0,645-1,055	0,124
Q3	1,627	1,304-2,030	<0.001	1,024	0,820-1,278	0,832	0,972	0,784-1,205	0,794
Q4	4,045	3,267-5,008	<0.001	1,784	1,446-2,201	<0,001	1,354	1,086-1,689	0,008
**ICC**									
Osm (contínua)	1,108	1,092-1,123	<0,001	1,044	1,031-1,056	<0,001	1,025	1,013-1,037	<0,001
Osm (categórica)	Valor de p para tendência < 0,001			Valor de p para tendência < 0,001			Valor de p para tendência < 0,001		
Q1	—	—		—	—		—	—	
Q2	1,245	1,009-1,537	0.041	1.029	0.832, 1.273	0.791	1.095	0.884, 1.356	0.404
Q3	1,998	1,617-2,468	<0,001	1,145	0,914-1,434	0.237	1.169	0.929, 1.471	0.181
Q4	4,345	3,590-5,259	<0,001	1,724	1,429-2,081	<0,001	1,459	1,207-1,764	<0,001
**Angina**									
Osm (contínua)	1,080	1,062-1,098	<0,001	1,028	1,011-1,045	0,001	1,011	0,995, 1,027	0,169
Osm (categórica)	Valor de p para tendência < 0,001			Valor de p para tendência = 0,002			Valor de p para tendência = 0,103		
Q1	—	—		—	—		—	—	
Q2	0,929	0,724-1,192	0,559	0,8	0,627-1,021	0,073	0,844	0,659-1,080	0,176
Q3	1,460	1,124-1,896	0,005	0,938	0,722-1,219	0,629	0,952	0,727-1,247	0,72
Q4	2,979	2,350-3,775	<0,001	1,396	1,104-1,765	0,006	1,188	0,924-1,527	0,177
**Infarto do miocárdio**									
Osm (contínua)	1,093	1,078-1,109	<0,001	1,035	1,021-1,049	<0,001	1,02	1,007-1,034	0,002
Osm (categórica)	Valor de p para tendência < 0,001			Valor de p para tendência < 0,001			Valor de p para tendência = 0,006		
Q1	—	—		—	—		—	—	
Q2	1,068	0,892-1,279	0,471	0,892	0,740-1,074	0,224	0,952	0,785-1,155	0,618
Q3	1,808	1,480-2,209	<0,001	1,099	0,892-1,355	0,371	1,145	0,931-1,408	0,197
Q4	3,46	2,849-4,201	<0,001	1,495	1,224-1,826	<0,001	1,315	1,072-1,614	0,009

Modelo 1: não ajustado. Modelo 2: ajustado por idade, sexo e raça/etnia. Modelo 3: ajustado por idade, sexo, raça/etnia, histórico de hipertensão, PAS, PAD, diabetes melito, triglicerídeos, LDL-C, BUN, glicose plasmática e IMC. BUN: nitrogênio ureico; DAC: doença arterial coronariana; ICC: insuficiência cardíaca congestiva; IMC: índice de massa corporal; PAD: pressão arterial diastólica; PAS: pressão arterial sistólica; LDL-C: colesterol de lipoproteína de baixa densidade; OR: odds ratio; Osm: osmolalidade plasmática.

**Figura 2:**
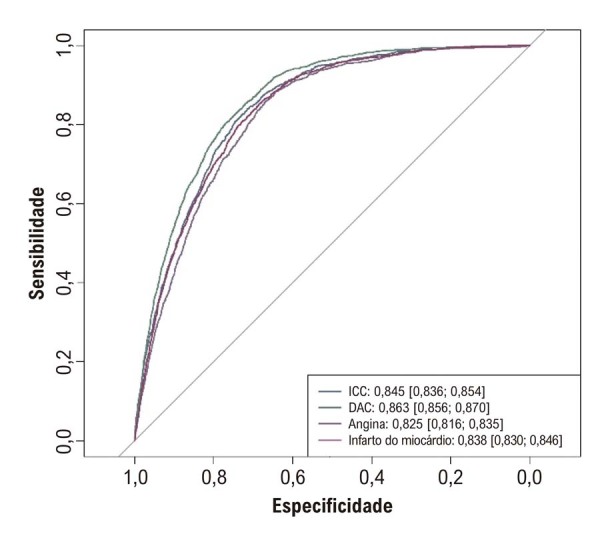
– Análise da curva receiver operating characteristic do Modelo 3. DAC: doença arterial coronariana; ICC: insuficiência cardíaca congestiva.

A análise ROC do Modelo 3 está na Figura 2 . O melhor desempenho ocorreu para DAC (AUC = 0,863). Os valores de pseudo-R^2^ de McFadden para o Modelo 3 foram 0,204 (ICC), 0,240 (DAC), 0,172 (angina) e 0,195 (infarto do miocárdio). Quando Osm foi analisada categoricamente, Q4 apresentou riscos maiores de ICC, DAC e infarto do que Q1. O risco de angina também foi maior em Q4 nos Modelos 1 e 2, mas não no modelo totalmente ajustado ( Tabela 2 ).

**Figura 3:**
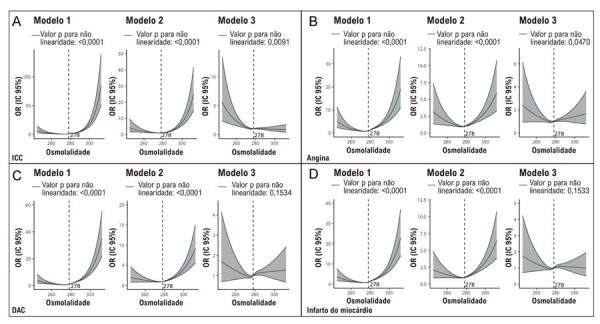
– Resultados da análise restricted cubic spline. A) Associações entre Osm e ICC. B) Associações entre Osm e angina. C) Associações entre Osm e DAC. D) Associações entre Osm e infarto do miocárdio. DAC: doença arterial coronariana; ICC: insuficiência cardíaca congestiva; OR: odds ratio; Osm: osmolalidade plasmática.

As análises com RCS ( Figura 3 ) identificaram relações não lineares significativas entre Osm e ICC, DAC, angina e infarto do miocárdio nos modelos não ajustado e parcialmente ajustado (valor p para não linearidade < 0,0001). No modelo totalmente ajustado, as associações não lineares para DAC e infarto deixaram de ser evidentes, enquanto as relações em U para ICC e angina persistiram.

**Figura 4:**
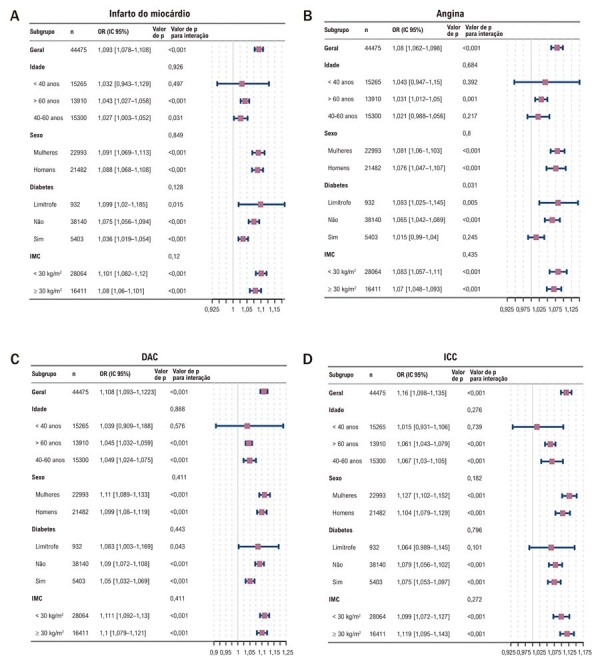
– Resultados da análise por subgrupos. A) Infarto do miocárdio; B) Angina; C) DAC; D) ICC. DAC: doença arterial coronariana; DM: diabetes melito; ICC: insuficiência cardíaca congestiva; OR: odds ratio.

As análises de subgrupos por idade, sexo, status de diabetes e IMC ( Figura 4 ) mostraram associações positivas entre Osm e ICC, DAC e infarto do miocárdio entre participantes > 40 anos, com achados consistentes entre subgrupos de sexo, diabetes e obesidade. Para angina, Osm associou-se positivamente entre indivíduos > 60 anos, com ou sem diabetes e com ou sem obesidade. Observou-se interação entre Osm e diabetes (p = 0,031).

Conforme resumido na Figura Central , a regressão logística evidenciou uma associação dose–resposta significativa entre Osm e o risco de DCV (ICC, DAC, angina e infarto do miocárdio), enquanto as análises por RCS mostraram associações em U especificamente para ICC e angina.

## Discussão

A partir dos dados do NHANES, examinamos a associação entre Osm e DCV. Entre 44.475 participantes, predominaram mulheres, indivíduos brancos não hispânicos e pessoas ≤ 60 anos. Valores mais altos de Osm associaram-se a risco significativamente maior de DCV. Após o ajuste completo, a associação com angina deixou de ser significativa. As análises com RCS identificaram uma associação não linear: padrão em U para ICC e angina em todos os modelos, com menor risco em torno de 278 mmol/kg. As análises de subgrupos sustentaram esses achados de forma consistente.

Estudos prévios vincularam Osm ao prognóstico em diferentes populações.^18 - 25^ Na diabetes, tanto Osm baixa quanto elevada associaram-se a maior mortalidade por todas as causas, sugerindo relação em U.^12^ Kaya et al. relataram faixa normal de Osm de 275-295 mOsm/kg e faixa “ótima” de 292-293 mOsm/kg; valores fora desse intervalo associaram-se a piores desfechos,^18^ alinhando-se ao padrão em U observado para ICC neste estudo. Osm elevada frequentemente reflete desidratação, hipernatremia ou hiperglicemia; variações ≤ 1% podem aumentar a liberação de arginina vasopressina (AVP).^26^ Através dos receptores V1A, a AVP promove vasoconstrição periférica e estimulação miocárdica; pelos receptores renais V2, induz retenção hídrica,^27^ contribuindo para ICC. Osm baixa tem sido associada a desregulação neuro-hormonal, descompensação e fadiga,^28 , 29^ favorecendo retenção de fluidos e aumento da pré-carga, o que pode deflagrar ou agravar ICC.

Observamos também associação em U entre Osm e angina. Embora atenuada após o ajuste completo, ao nosso conhecimento essa relação não havia sido descrita. Estresse hipotônico aumenta volume celular e proliferação — sobretudo de células musculares lisas vasculares — processos implicados em DCV.^30 , 31^ Também influencia a agregação plaquetária e eleva o volume plaquetário médio, marcador de ativação plaquetária.^32 , 33^ Por outro lado, sódio elevado pode estimular a liberação endotelial de fator de von Willebrand e promover hipercoagulabilidade.^34^ Condições associadas à hipernatremia — como desidratação, idade avançada, diabetes, dietas ricas em sal e terapia hiperosmolar — podem predispor à trombose.^35^

As análises de subgrupos indicaram associações amplamente consistentes entre Osm e desfechos de DCV em categorias de idade, sexo, status de diabetes e IMC. Para angina, as associações positivas foram evidentes em adultos > 60 anos, com ou sem diabetes, e com ou sem obesidade. Detectou-se interação significativa entre Osm e diabetes. Uma explicação possível é que pessoas com diabetes estabelecido recebam manejo mais intensivo de fatores de risco (p. ex., controle glicêmico, pressórico e lipídico), enquanto indivíduos sem diabetes ou com pré-diabetes podem não receber intervenções comparáveis, o que influenciaria o risco de angina.

Este estudo faz um exame preliminar da associação entre Osm e DCV, oferecendo uma nova lente para investigar risco cardiovascular. Osm elevada pode atuar como sinal de alerta precoce, especialmente em grupos de alto risco. Incorporar o monitoramento de Osm na avaliação e no manejo de DCV pode ajudar a melhorar o prognóstico e reduzir o risco, e Osm pode integrar programas de rastreio precoce e estratificação de risco.

Algumas limitações merecem consideração. Primeiro, o delineamento transversal impede inferência causal; estudos prospectivos de coorte são necessários para confirmação. Segundo, apesar do grande tamanho amostral, a estratificação limitada de subpopulações específicas pode restringir a generalização. Terceiro, o NHANES oferece um conjunto limitado de variáveis, e fatores-chave — como ecocardiografia, angiografia coronariana, função renal (taxa de filtração glomerular estimada), uso de diuréticos e estado de hidratação — não estavam disponíveis. Quarto, por se tratar majoritariamente de americanos brancos não hispânicos, os achados podem não se aplicar a outras regiões, como África e Ásia. Potenciais vieses incluem viés de seleção decorrente de participação domiciliar e voluntária; viés de informação, pois Osm foi calculada indiretamente e não medida por osmometria; viés de autorrelato, já que os desfechos — ICC, DAC, angina e infarto do miocárdio — vieram de questionários e não foram adjudicados clinicamente; e possível viés de publicação devido ao destaque dado a achados positivos, incluindo associações que se atenuaram até a não significância após o ajuste completo.

## Conclusão

Osm elevada associa-se de forma significativa ao risco de DCV, sugerindo que Osm pode ser um biomarcador útil para avaliação de risco e manejo clínico. No entanto, esses achados derivam de dados transversais e requerem validação em estudos de coorte prospectivos.
