# Supplementary material for: Associação da Osmolalidade Plasmática com o Risco de Doença Cardiovascular: Evidências do Banco de Dados do National Health and Nutrition Examination Survey
Source: Arq Bras Cardiol. 2025 Dec 8;122(11):e20250176. [Article in Portuguese] doi: 10.36660/abc.20250176 (PMC12711222; doi:10.36660/abc.20250176)
Supplement: Translation

# Translation: Association of Plasma Osmolality with Cardiovascular Disease Risk: Evidence from the National Health and Nutrition Examination Survey Database

**DOI:** 10.36660/abc.20250176i

**Authors:** Feng Gao, Yicheng Shi, Yi Chen, Shu Lu

**Affiliations:** 1 China Nanjing University of Traditional Chinese Medicine Affiliated Wuxi Hospital – Cardiovascular, Wuxi – China

**Keywords:** Cardiovascular Diseases, Heart Failure, Coronary Disease, Myocardial Infarction

## Abstract

**Background:**

Cardiovascular disease (CVD) remains the world’s leading cause of death. Because of its substantial public health burden, further research is needed to define risk factors that are both modifiable and measurable.

**Objective:**

We examined the association between plasma osmolality (Osm) and CVDs — namely, heart failure, coronary heart disease, angina, and myocardial infarction — using data from the National Health and Nutrition Examination Survey (NHANES).

**Methods:**

We analyzed nine NHANES cycles (1999-2018). Associations between Osm and CVD were assessed with weighted logistic regression. Odds ratios (ORs) and 95% CIs were estimated, and subgroup analyses were conducted. Restricted cubic spline (RCS) models were used to evaluate potential nonlinear relationships. Statistical significance was set at p < 0.05.

**Results:**

Higher Osm was associated with increased odds of heart failure, coronary heart disease, angina, and myocardial infarction in unadjusted and partially adjusted models. After full adjustment, the associations remained significant for all outcomes except angina. RCS analyses indicated a significant U-shaped relationship for heart failure and angina across all models, with the lowest risk around 278 mmol/kg. Subgroup analyses were consistent with the primary results.

**Conclusion:**

Increased Osm is significantly associated with CVD risk. Osm may serve as a useful biomarker for assessment and management of CVD risk in clinical practice, pending confirmation in prospective cohort studies.

## Introduction

Cardiovascular disease (CVD) encompasses conditions affecting the heart and blood vessels, including coronary heart disease (CHD), hypertension, and heart failure (HF).^1 - 5^The World Health Organization identifies CVD as the leading cause of death globally, accounting for approximately 17.9 million deaths each year,^6^with profound impacts on patients and families and a substantial economic burden on society. Despite advances in pharmacological, interventional, and surgical therapies, substantial limitations remain — particularly in risk stratification and personalized treatment.^5 , 7 - 9^Consequently, there is an urgent need for research to deepen our understanding of CVD risk factors.

Plasma osmolality (Osm) regulates water balance across intra- vs. extracellular and intra- vs. extravascular compartments and is largely determined by Na^+^, K^+^, and Cl^–^. $\text{Osm}=2\times {\mathrm{Na}}^{+}+\text{glucose/18 + blood urea nitrogen (BUN)2,8}$ , with a normal range of 275-295 mOsm/kg.^10^Measuring and monitoring Osm is essential in the assessment and management of numerous conditions. Alterations in Osm are closely linked to the course of disorders such as hyponatremia, hypernatremia, hyperglycemia, osteoarthritis, nephropathy, and chronic obstructive pulmonary.^11^Emerging evidence also suggests a relationship between Osm and cardiovascular outcomes. In diabetic patients, both low and high Osm were associated with increased all-cause mortality, indicating a U-shaped association.^12^The blood urea nitrogen-to-albumin ratio (BAR) — with BUN as a major Osm component — predicts mortality in severe CHD and CHF.^13 , 14^Osm is furthermore a key prognostic indicator in CHF, correlating with 28-day all-cause mortality.^15^However, most prior work has emphasized prognosis among patients with established CVD, and comprehensive evaluations of Osm and CVD risk in the overall population remain limited.

By using data from the National Health and Nutrition Examination Survey (NHANES), we examined the association between Osm and the risk of CHF, CHD, angina, and myocardial infarction, with the goal of informing clinical risk assessment.

## Methods

### Study participants

We used data from the NHANES (https://www.cdc.gov/nchs/nhanes/index.html), a nationally representative program that assesses the health and nutritional status of the U.S. population through continuous, multistage probability sampling.^16^Home interviews were followed by physical and laboratory examinations conducted at Mobile Examination Centers (MECs). All NHANES protocols were approved by the National Center for Health Statistics Ethics Review Board, and written informed consent was obtained from all participants. All analyses adhered to NHANES guidelines and regulations.

We analyzed the most recent nine NHANES cycles (1999-2018), comprising 91,351 participants. Individuals with missing Osm measurements or missing CVD questionnaire data were excluded.

### Exposure and outcomes

Osm was measured at MECs using the NHANES variable LBXSOSSI and ranged from 201 to 323 mmol/kg. Participants were categorized into quartiles (Q1-Q4), with Q1 denoting the lowest and Q4 the highest Osm values.

The primary outcomes were the prevalences of CHF, CHD, angina, and myocardial infarction, defined by self-reported questionnaire items: MCQ160b (“Ever told had CHF”), MCQ160c (“Ever told you had CHD”), MCQ160d (“Ever told you had angina/angina pectoris”), and MCQ160e (“Ever told you had heart attack”). Respondents answering “Yes” were classified as having the condition; those answering “No” were classified as not having the condition.

### Covariates

Following Bays et al.,^17^we adjusted for age; sex; race/ethnicity (Mexican American, other Hispanic, non-Hispanic White, non-Hispanic Black, other); histories of hypertension and diabetes; systolic (SBP) and diastolic blood pressure (DBP); smoking status; body mass index (BMI); triglycerides (TG); low-density lipoprotein cholesterol (LDL-C); BUN; and blood glucose. Demographic characteristics and histories of hypertension, diabetes, and smoking were obtained by questionnaire. SBP, DBP, and BMI were derived from examination data collected at MECs, and TG, LDL-C, BUN, and glucose were obtained from MEC laboratory data. Smoking status was categorized as “Every day,” “Someday,” or “Not at all.” TG was dichotomized as ≤ 1.70 vs > 1.70 mmol/L, and LDL-C as ≤ 3.37 vs > 3.37 mmol/L. To limit bias from missingness, variables with ≤ 20% missing data underwent multiple imputation; for variables with > 20% missingness, categories were created and included as dummy variables in the models.

### Statistical analysis

Normality was assessed with the Shapiro-Wilk test. Because all continuous variables were nonnormally distributed, they are reported as medians with interquartile ranges and compared using the Mann-Whitney *U* test. Categorical variables are presented as counts (n) and percentages (%) and were compared using the chi-square test.

Weighted logistic regression (WLR) was used to estimate associations between exposure and outcomes, with covariates added sequentially. Model 1 was unadjusted; Model 2 adjusted for age, sex, and race; Model 3 additionally adjusted for hypertension, diabetes, smoking, SBP, DBP, BMI, TG, LDL-C, BUN, and blood glucose. To evaluate the discriminative performance of Model 3, we generated receiver operating characteristic (ROC) curves and calculated the area under the curve (AUC) and McFadden’s pseudo-R^2^. To mitigate multicollinearity, we computed variance inflation factors (VIFs) and excluded variables with VIF > 5. Restricted cubic spline (RCS) models assessed potential nonlinear relationships. Subgroup analyses were then performed and displayed as forest plots. Analyses were conducted in R version 4.4.2, with two-sided p < 0.05 considered statistically significant.

## Results

A total of 44,475 participants met the eligibility criteria ( Figure 1 ). Because all continuous variables were nonnormally distributed, the Mann-Whitney *U* test was used. Baseline characteristics are shown in Table 1 and indicate a predominance of females, non-Hispanic White participants, and individuals ≤ 60 years. Compared with Q1 (lowest Osm), Q4 (highest Osm) included more males and older adults (> 60 years) and showed higher prevalences of hypertension, diabetes, CHF, CHD, angina, and heart attack, as well as higher BMI, SBP, BUN, and glucose levels.

**Figure 1:** – Study enrollment flow diagram. CVD: cardiovascular disease; CHD: coronary heart disease; CHF: congestive heart failure; NHANES: National Health and Nutrition Examination Survey; Osm: plasma osmolality.

**Table 1:** – Baseline characteristics of participants by Osm quartile

Caracteristic	Total	Q1	Q2	Q3	Q4	p-value
**N (weighted)**	206,406,933	58,579,788	53,539,459	58,183,076	36,104,609	
**BMI**	28 [24, 32]	27 [24, 32]	27 [24, 32]	28 [24, 32]	29 [25, 33]	<0.001***
**Hypertension, n (%)**	15,455 (31)	3,205 (25)	3,112 (26)	4,552 (32)	4,586 (46)	<0.001***
**SBP, mmHg**	120 [112, 132]	118 [110, 128]	118 [110, 130]	122 [112, 132]	126 [116, 136]	<0.001***
**DBP, mmHg**	71 [64, 78]	70 [64, 78]	71 [64, 78]	72 [64, 78]	71 [64, 78]	0.028*
**Sex (%)**						<0.001***
Male	21,482 (48)	4,562 (37)	5,391 (49)	6,753 (55)	4,776 (54)	
Female	22,993 (52)	7,895 (63)	5,440 (51)	5,595 (45)	4,063 (46)	
**Age group (%)**						<0.001***
< 40 years	15,265 (37)	6,213 (49)	4,379 (42)	3,460 (32)	1,213 (19)	
40-60 years	15,300 (40)	4,067 (38)	4,020 (42)	4,502 (41)	2,711 (37)	
> 60 years	13,910 (23)	2,177 (13)	2,432 (16)	4,386 (27)	4,915 (45)	
**Race/Ethnicity (%)**						<0.001***
Mexican American	7,499 (8.3)	2,238 (8.9)	1,880 (8.7)	2,030 (7.8)	1,351 (7.7)	
Other Hispanic	3,753 (5.4)	981 (5.5)	987 (5.8)	1,045 (5.1)	740 (5.2)	
Non-Hispanic Black	9,069 (11)	2,639 (12)	2,195 (10)	2,402 (9.7)	1,833 (10)	
Non-Hispanic White	19,835 (68)	5,397 (66)	4,711 (68)	5,678 (70)	4,049 (69)	
Other	4,319 (7.1)	1,202 (7.2)	1,058 (7.1)	1,193 (6.9)	866 (7.2)	
**Diabetes status (%)**						<0.001***
Yes	5,403 (9.0)	697 (4.3)	816 (5.8)	1,532 (9.3)	2,358 (21)	
No	38,140 (89)	11,581 (94)	9,827 (93)	10,532 (89)	6,200 (76)	
Borderline	932 (1.9)	179 (1.3)	188 (1.5)	284 (2.0)	281 (3.2)	
**Smoking status (%)**						<0.001***
Every day	7,531 (17)	2,505 (22)	1,985 (18)	1,968 (15)	1,073 (12)	
Missing	24,275 (54)	6,917 (53)	6,033 (56)	6,648 (54)	4,677 (54)	
Not at all	10,939 (25)	2,491 (21)	2,321 (22)	3,293 (27)	2,834 (31)	
Some days	1,730 (3.7)	544 (4.2)	492 (4.2)	439 (3.4)	255 (2.8)	
**Triglycerides, n (%)**						0.017*
≤ 1.70 mmol/L	15,482 (35)	4,199 (34)	3,880 (35)	4,308 (35)	3,095 (36)	
> 1.70 mmol/L	5,877 (13)	1,618 (13)	1,366 (12)	1,636 (13)	1,257 (14)	
Missing	23,116 (52)	6,640 (54)	5,585 (52)	6,404 (52)	4,487 (50)	
**LDL-C (%)**						<0.001***
≤ 3.37 mmol/L	14,486 (33)	3,990 (32)	3,526 (32)	3,913 (32)	3,057 (35)	
> 3.37 mmol/L	6,271 (14)	1,643 (13)	1,598 (14)	1,876 (15)	1,154 (14)	
Missing	23,718 (53)	6,824 (55)	5,707 (54)	6,559 (53)	4,628 (51)	
**BUN, mg/dL**	13.0 [10.0, 16.0]	11.0 [8.0, 13.0]	12.0 [10.0, 15.0]	14.0 [11.0, 17.0]	16.0 [13.0, 21.0]	<0.001***
**Plasma glucose, mmol/L**	5.05 [4.72, 5.61]	4.88 [4.55, 5.27]	5.00 [4.66, 5.44]	5.16 [4.77, 5.72]	5.50 [5.00, 6.61]	<0.001***
**CHF, n (%)**	1,397 (2.3)	216 (1.4)	200 (1.3)	363 (2.2)	618 (5.3)	<0.001***
**CHD, n (%)**	1,815 (3.4)	275 (1.8)	294 (2.3)	511 (3.6)	735 (7.5)	<0.001***
**Angina, n (%)**	1,218 (2.3)	215 (1.6)	198 (1.5)	355 (2.3)	450 (4.7)	<0.001***
**Myocardial infarction, n (%)**	1,856 (3.2)	294 (2.0)	292 (2.1)	555 (3.5)	715 (6.5)	<0.001***

Data are presented as median [Q1, Q3] or n (%). Quartiles of plasma osmolality (mOsm/kg): Q1 201-275; Q2 275-278; Q3 278-282; Q4 282-323. N (weighted) indicates weighted sample size. P-value thresholds: *p < 0.05; **p < 0.01; ***p < 0.001. BMI: body mass index; BUN: blood urea nitrogen; CHD: coronary heart disease; CHF: congestive heart failure; DBP: diastolic blood pressure; LDL-C: low-density lipoprotein cholesterol; Osm: plasma osmolality; SBP: systolic blood pressure.

WLR analyses ( Table 2 ) showed that higher Osm was associated with substantially increased CVD risk in unadjusted, partially adjusted, and fully adjusted models. When modeled continuously, higher Osm was significantly associated with greater risks of CHF, CHD, and myocardial infarction across all three models. Angina showed the same pattern in unadjusted and partially adjusted models, but the association was no longer significant after full adjustment ( Table 2 ).

**Table 2:** – Association between Osm and cardiovascular outcomes

Caracteristic	Model 1			Model 2			Model 3		
	OR	95% CI	p-value	OR	95% CI	p-value	OR	95% CI	p-value
**CHF**									
Plasma osmolality (continuous)	1.116	1.097, 1.135	<0.001	1.059	1.042, 1.076	<0.001	1.033	1.017, 1.049	<0.001
Plasma osmolality (categorical)	p-value for trend < 0.001			p-value for trend < 0.001			p-value for trend = 0.004		
Q1	—	—		—	—		—	—	
Q2	0.946	0.745, 1.201	0.645	0.816	0.642, 1.037	0.096	0.825	0.645, 1.055	0.124
Q3	1.627	1.304, 2.030	<0.001	1.024	0.820, 1.278	0.832	0.972	0.784, 1.205	0.794
Q4	4.045	3.267, 5.008	<0.001	1.784	1.446, 2.201	<0.001	1.354	1.086, 1.689	0.008
**CHD**									
Osm (continuous)	1.108	1.092, 1.123	<0.001	1.044	1.031, 1.056	<0.001	1.025	1.013, 1.037	<0.001
Osm (categorical)	p-value for trend < 0.001			p-value for trend < 0.001			p-value for trend < 0.001		
Q1	—	—		—	—		—	—	
Q2	1.245	1.009, 1.537	0.041	1.029	0.832, 1.273	0.791	1.095	0.884, 1.356	0.404
Q3	1.998	1.617, 2.468	<0.001	1.145	0.914, 1.434	0.237	1.169	0.929, 1.471	0.181
Q4	4.345	3.590, 5.259	<0.001	1.724	1.429, 2.081	<0.001	1.459	1.207, 1.764	<0.001
**Angina**									
Osm (continuous)	1.08	1.062, 1.098	<0.001	1.028	1.011, 1.045	0.001	1.011	0.995, 1.027	0.169
Osm (categorical)	p-value for trend < 0.001			p-value for trend = 0.002			p-value for trend = 0.103		
Q1	—	—		—	—		—	—	
Q2	0.929	0.724, 1.192	0.559	0.8	0.627, 1.021	0.073	0.844	0.659, 1.080	0.176
Q3	1.46	1.124, 1.896	0.005	0.938	0.722, 1.219	0.629	0.952	0.727, 1.247	0.72
Q4	2.979	2.350, 3.775	<0.001	1.396	1.104, 1.765	0.006	1.188	0.924, 1.527	0.177
**Myocardial infarction**									
Osm (continuous)	1.093	1.078, 1.109	<0.001	1.035	1.021, 1.049	<0.001	1.02	1.007, 1.034	0.002
Osm (categorical)	p-value for trend < 0.001			p-value for trend < 0.001			p-value for trend = 0.006		
Q1	—	—		—	—		—	—	
Q2	1.068	0.892, 1.279	0.471	0.892	0.740, 1.074	0.224	0.952	0.785, 1.155	0.618
Q3	1.808	1.480, 2.209	<0.001	1.099	0.892, 1.355	0.371	1.145	0.931, 1.408	0.197
Q4	3.46	2.849, 4.201	<0.001	1.495	1.224, 1.826	<0.001	1.315	1.072, 1.614	0.009

Model 1: unadjusted. Model 2: adjusted for age, sex, and race/ethnicity. Model 3: adjusted for age, sex, race/ethnicity, hypertension history, SBP, DBP, diabetes mellitus, triglycerides, LDL-C, BUN, plasma glucose, and BMI. BMI: body mass index; BUN: blood urea nitrogen; CHD: coronary heart disease; CHF: congestive heart failure; DBP: diastolic blood pressure; LDL-C: low-density lipoprotein cholesterol; OR: odds ratio; Osm: plasma osmolality; SBP: systolic blood pressure.

ROC analysis for Model 3 is shown in Figure 2 . Model 3 performed best for CHD (AUC = 0.863). McFadden’s pseudo-R^2^values for Model 3 were 0.204 for CHF, 0.240 for CHD, 0.172 for angina, and 0.195 for myocardial infarction. When Osm was analyzed categorically, Q4 had higher risks of CHF, CHD, and myocardial infarction than Q1. The risk of angina was also higher in Q4 for Models 1 and 2, but not in the fully adjusted model ( Table 2 ).

**Figure 2:** – Receiver operating characteristic curve analysis of Model 3. CHF: congestive heart failure; CHD: coronary heart disease.

RCS analyses ( Figure 3 ) identified significant nonlinear relationships between Osm and CHF, CHD, angina, and myocardial infarction in unadjusted and partially adjusted models (p for nonlinearity < 0.0001). In the fully adjusted model, the nonlinear associations for CHD and myocardial infarction were no longer evident, whereas the nonlinear relationships for CHF and angina persisted.

**Figure 3:** – Restricted cubic spline analysis results. A) Associations between OSM and CHF. B) Associations between osmolality and angina. C) Associations between osmolality and CHD. D) Associations between osmolality and myocardial infarction. CHF: congestive heart failure, CHD: coronary heart disease; OR: odds ratio; Osm: plasma osmolality.

Subgroup analyses by age, sex, diabetes status, and BMI are presented in Figure 4 . Among participants > 40 years, Osm was positively associated with incident CHF, CHD, and myocardial infarction, with consistent findings across sex, diabetes, and obesity subgroups. For angina, Osm was positively associated among individuals > 60 years, those with or without diabetes, and those with or without obesity. An interaction between Osm and diabetes was observed (p = 0.031).

**Figure 4:** – Results of subgroup analysis. A) MI; B) Angina; C) CHD. D) CHF. CHF: congestive heart failure; CHD: coronary heart disease; DM: diabetes mellitus; OR: odds ratio.

As summarized in Central Illustration , logistic regression demonstrated a significant dose-response association between Osm and CVD risk (CHF, CHD, angina, and myocardial infarction), whereas RCS analyses showed significant U-shaped associations specifically for CHF and angina.

## Discussion

By using NHANES data, we examined the association between Osm and CVD. Among 44,475 participants, women, non-Hispanic White individuals, and those ≤ 60 years predominated. Higher Osm was associated with a significantly greater CVD risk. After full adjustment, the association with angina was no longer significant. RCS analyses identified a nonlinear association: a U-shaped pattern for CHF and angina across all models, with the lowest risk around 278 mmol/kg. Subgroup analyses consistently supported these findings.

Prior studies have linked Osm to prognosis across diverse patient groups.^18 - 25^In diabetes, both low and high Osm have been associated with higher all-cause mortality, indicating a U-shaped relationship.^12^Kaya et al. reported a normal Osm range of 275-295 mOsm/kg and an optimal range of 292-293 mOsm/kg, with values outside this range associated with worse outcomes,^18^which aligns with the U-shaped pattern observed for CHF in our study. Increased Osm often reflects dehydration, hypernatremia, or hyperglycemia; even a ≤ 1% change can increase arginine vasopressin (AVP) release.^26^Via V1A receptors, AVP promotes peripheral vasoconstriction and myocardial stimulation, and via renal V2 receptors it induces water retention,^27^thereby contributing to CHF. Low Osm has been linked to neurohormonal dysregulation, decompensation, and fatigue,^28 , 29^which may promote fluid retention and increased preload, potentially triggering or worsening CHF.

We also observed a U-shaped association between Osm and angina. Although this association attenuated after full adjustment, to our knowledge it has not been previously described. Hypotonic stress increases cell volume and proliferation — particularly of vascular smooth-muscle cells — processes implicated in CVD.^30 , 31^It also influences platelet aggregation and increases mean platelet volume, a marker of platelet activation.^32 , 33^Conversely, high sodium levels can stimulate endothelial release of von Willebrand factor and promote hypercoagulability.^34^Conditions associated with hypernatremia — including dehydration, older age, diabetes, high-salt diets, and hyperosmolar therapy — may predispose to thrombosis.^35^

Subgroup analyses indicated broadly consistent associations between Osm and CVD outcomes across age, sex, diabetes status, and BMI categories. For angina, positive associations were evident among adults > 60 years and among individuals with or without diabetes or obesity. A significant interaction between Osm and diabetes was detected. One possible explanation is that people with established diabetes may receive more intensive risk-factor management (e.g., glycemic, blood-pressure, and lipid control), whereas those without diabetes or with prediabetes may not receive comparable interventions, which could influence angina risk.

This study offers a preliminary examination of the association between Osm and CVD, providing a new lens for investigating CVD risk. Increased Osm may serve as an early warning signal, particularly in high-risk groups. Incorporating Osm monitoring into CVD assessment and management could help improve prognosis and reduce risk, and Osm could be incorporated into early screening and risk-stratification programs.

Several limitations merit consideration. First, the cross-sectional design precludes causal inference; prospective cohort studies are needed for confirmation. Second, although the sample was large, limited stratification of specific subpopulations may restrict generalizability. Third, NHANES provides a constrained set of variables, and key factors — such as echocardiography, coronary angiography, renal function (estimated glomerular filtration rate), diuretic use, and hydration status — were unavailable. Fourth, because the population was predominantly non-Hispanic White Americans, the findings may not generalize to other regions, such as Africa and Asia. Potential biases include selection bias from household, voluntary participation; information bias, as Osm was calculated indirectly rather than measured by osmometry; self-report bias, because outcomes — namely, CHF, CHD, angina, myocardial infarction — were questionnaire-based rather than clinically adjudicated; and possible publication bias due to emphasis on positive findings, including associations that attenuated to nonsignificance after full adjustment.

## Conclusion

Increased Osm is significantly associated with CVD risk, suggesting that Osm may be a useful biomarker for risk assessment and clinical management. These findings, however, derive from cross-sectional data and require validation in prospective cohort studies.
